# Mixed-sex clusters on grass blades: breeding strategy of the ornate dog tick, *Dermacentor reticulatus*

**DOI:** 10.1186/s13071-024-06129-4

**Published:** 2024-02-09

**Authors:** Dagmara Wężyk, Wiktoria Romanek, Wiktoria Małaszewicz, Jerzy M. Behnke, Anna Bajer

**Affiliations:** 1 Department of Eco-Epidemiology of Parasitic Diseases, Institute of Developmental Biology and Biomedical Sciences, Faculty of Biology, University of Warsaw, Miecznikowa 1, 02-096 Warsaw, Poland; 2 School of Life Sciences, University of Nottingham, University Park, Nottingham, NG7 2RD UK

**Keywords:** Behaviour, Ticks, *Dermacentor reticulatus*, Aggregations, Assembly, Single-sex clusters, Mixed-sex clusters

## Abstract

**Background:**

The ornate dog tick *Dermacentor reticulatus* is second only to the hard tick *Ixodes ricinus* in terms of importance as a vector of infectious organisms, especially of *Babesia canis*, the agent of canine babesiosis. Both the geographical range and local densities of *D. reticulatus* are steadily increasing in many regions of Europe. In the present study, we tested the hypothesis that *D. reticulatus* possesses an efficient breeding strategy that allows for a rapid increase in tick numbers and densities through the formation of mixed-sex clusters/aggregations while questing in the environment.

**Methods:**

An observational study was carried out in the spring of 2023, at three sites in two regions in Central and North-Eastern Poland, both characterised by high tick densities. At each site, a 400-m-long transect was inspected for questing ticks. All noted ticks were collected, and tick numbers and sexes per stem were recorded. Differences in tick distribution by site and sex were analysed statistically.

**Results:**

A total of 371 *D. reticulatus* (219 females, 152 males) ticks were collected from 270 grass stems over a combined 1200 m of transect. The majority of grass stems (74.4%) were occupied by just a single individual, with two-tick clusters the second most common category. The maximum number of *D. reticulatus* individuals observed on a single grass stem was six.

Mixed-sex clusters were significantly more common than single-sex clusters at all three sites. With study sites combined, mixed-sex clusters accounted for 17.4% (95% confidence limit [95% CL] 13.9–21.6%) of observations, while for multiple males and multiple females, the values were 2.6% (95% CL: 1.4–4.7%) and 5.6% (95% CL: 3.7–8.3%), respectively.

**Conclusions:**

Mixed-sex clusters of *D. reticulatus* ticks were significantly more common than single-sex clusters, which we hypothesise reflects an efficient, likely pheromone-mediated breeding strategy of this expansive tick species.

**Graphical Abstract:**

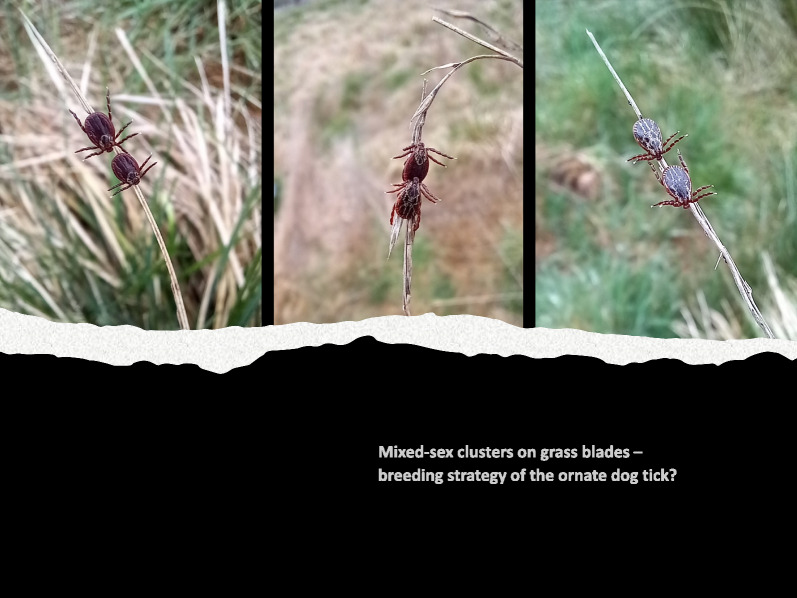

## Background

Ticks and tick-borne diseases pose an increasing threat to human and animal health worldwide. Consequently, interest in the role of ticks as vectors and their competence for transmitting multiple pathogens is increasing. Many aspects of the biology of the main tick vectors are understood in some detail [1–4], although tick behaviour has been less well studied. Authors of recent studies have reported a range of different activities of ticks under laboratory conditions [5] and identified significant differences in activity between ticks infected and uninfected with *Rickettsia* spp. [6]. However, tick behaviour under natural conditions is less well known [7, 8].

The ornate dog tick *Dermacentor reticulatus* is second only to the hard tick *Ixodes ricinus* in terms of importance as a vector of infectious organisms, including *Babesia canis* (a causative agent of canine babesiosis), tick-borne encephalitis virus (TBEV) and *Rickettsia raoulti* [9–12]. The geographical range of *D. reticulatus* is expanding in many regions of Europe [13–16], and its territorial expansion is accompanied by the emergence and spread of canine babesiosis in regions where previously dogs had been free of this disease [17–19]. Interestingly, in some areas where these two tick species occur sympatrically, *D. reticulatus* infestation on dogs and livestock is much more intense than infestation with *I. ricinus*, and mean tick density of *D. reticulatus* can be up to sevenfold higher than that of *I. ricinus* [20]. In a recent study, an extremely high population size of adult questing *D. reticulatus* was recorded in South-Eastern Poland, exceeding 344 ± 57.8 ticks per 100 m^2^ [21, 22]. Taking into account the ability of *D. reticulatus* ticks to expand their region of endemicity as well as to reach very high densities and infestation parameters, we hypothesised that *D. reticulatus* has to possess an extremely efficient breeding strategy to enable the observed rapid increase in tick numbers and densities.

*Dermacentor reticulatus* belongs to the Metastriata group of ixodid ticks that includes species of genera *Dermacentor*,* Amblyomma* and *Rhipicephalus*. This group of ticks invariably attain sexual maturity and mate solely on their hosts [1, 2, 23]. In contrast, *I. ricinus*, which belongs to the Prostriata group of ticks, can copulate also in the absence of a host. Following adult ecdysis, the germinal cells of metastriate male ticks become arrested as late prophase primary spermatocytes. Development proceeds only when the males find a host, usually 1 day after the adult male ticks attach to a host [2]. In our previous study, we observed that the body shape of *D. reticulatus* males collected from hosts changed despite little change in weight [20]. We found that these foraging males were filled with sperm containing mature spermatocytes, in contrast to questing males collected from vegetation, in which only primary spermatocytes were identified (Mierzejewska and Bębas, unpublished).

We hypothesised that for optimisation of their breeding strategy, males should accompany questing females in order to increase their chances of attachment to the same host individual, thereby enabling a blood meal for females and an opportunity for males to mature and copulate with engorged females. Thus, off-host mixed-sex clusters of two or more ticks should be more prevalent than single-sex assemblages.

## Methods

**Fig. 1:**
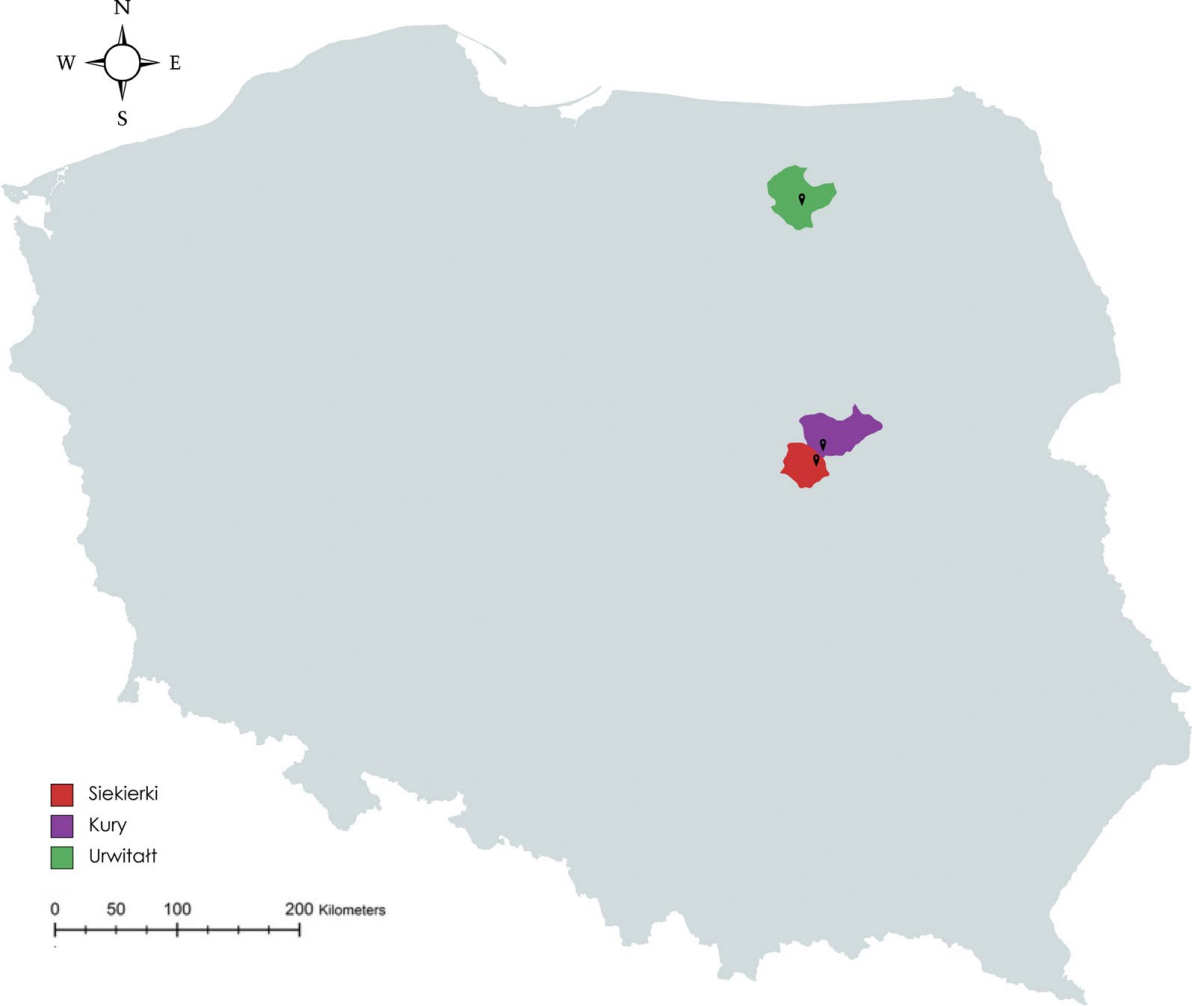
Map of Poland showing tick sighting locations

To test our hypothesis, we carried out an observational study in the spring of 2023, at three sites in two regions of Poland: two sites in Central Poland, both characterised by high tick densities (Kury and Siekierki–Warsaw in Mazovia (Masovia) Province and one site in North-Eastern Poland (Urwitałt in the Masurian [Mazury] Lake District) (Fig. 1). The study was conducted between 14 April and 5 May 2023, during the spring peak of *D. reticulatus* tick activity [17], when adults can be spotted easily on the tops of grass blades (Fig. 1). Each of four independent researcher(s) (AB, DW, WM, WR) walked along a 400-m-long transect at one of the three sites (traversing grassy areas in the 2 rural sites [Kury and Urwitałt] and 1 urban site [Siekierki–Warsaw]) looking for questing ticks on the tops of grass blades. The width of the transect was limited by the eyesight range of the observers/collectors. All noted ticks were collected, and tick numbers and sexes per stem were recorded.

## Statistical analysis

Data recorded from each of the three study sites included the number of individual grass stems with just a single male tick, with a single female, with multiple males, with multiple females or with mixed sexes. For these five data subsets, the number of records was converted to a percentage of the total observations from each site, and the percentages are given with 95% CLs (confidence limits) in the text and 95% CIs (95% confidence intervals) on figures; these percentages were calculated in bespoke software (see following paragraph) based on the tables of Rohlf and Sokal [24].

Data were analysed using maximum likelihood techniques based on log-linear analysis of contingency tables in the software package IBM SPSS (version 28; SPSS IBM Corp., Armonk, NY, USA). This approach is based on categorical values of factors of interest, which are used not only to fit hierarchical log-linear models to multidimensional cross-tabulations using an iterative proportional-fitting algorithm but also to detect associations between factors. We fitted a model with the following explanatory factors: site (3 levels, corresponding to Kury, Siekierki and Urwitałt); a single male tick (2 levels, present or absent); a single female tick (2 levels, present or absent); > 1 male tick but no females (2 levels, present or absent); > 1 female tick but no males (2 levels, present or absent); and mixed sexes (2 levels, present or absent). By employing the backward selection procedure in SPSS, we simplified each model until only significant terms remained. For each level of analysis in turn, beginning with the most complex model that involved all possible main effects and interactions, those combinations that did not contribute significantly to explaining variation in the data were eliminated in a stepwise fashion, beginning with the highest level interaction (backward selection procedure). A minimum sufficient model (MSM; Table 2) was then obtained, for which the likelihood ratio of Chi-square (*χ*^2^) was not significant, indicating that the model was sufficient in explaining the data. The importance of each term in the final model was assessed by the probability that its exclusion would alter the model significantly, and these values are given in the text, assessed by a likelihood ratio test between models with and without each term of interest.

## Results

**Fig. 2:**
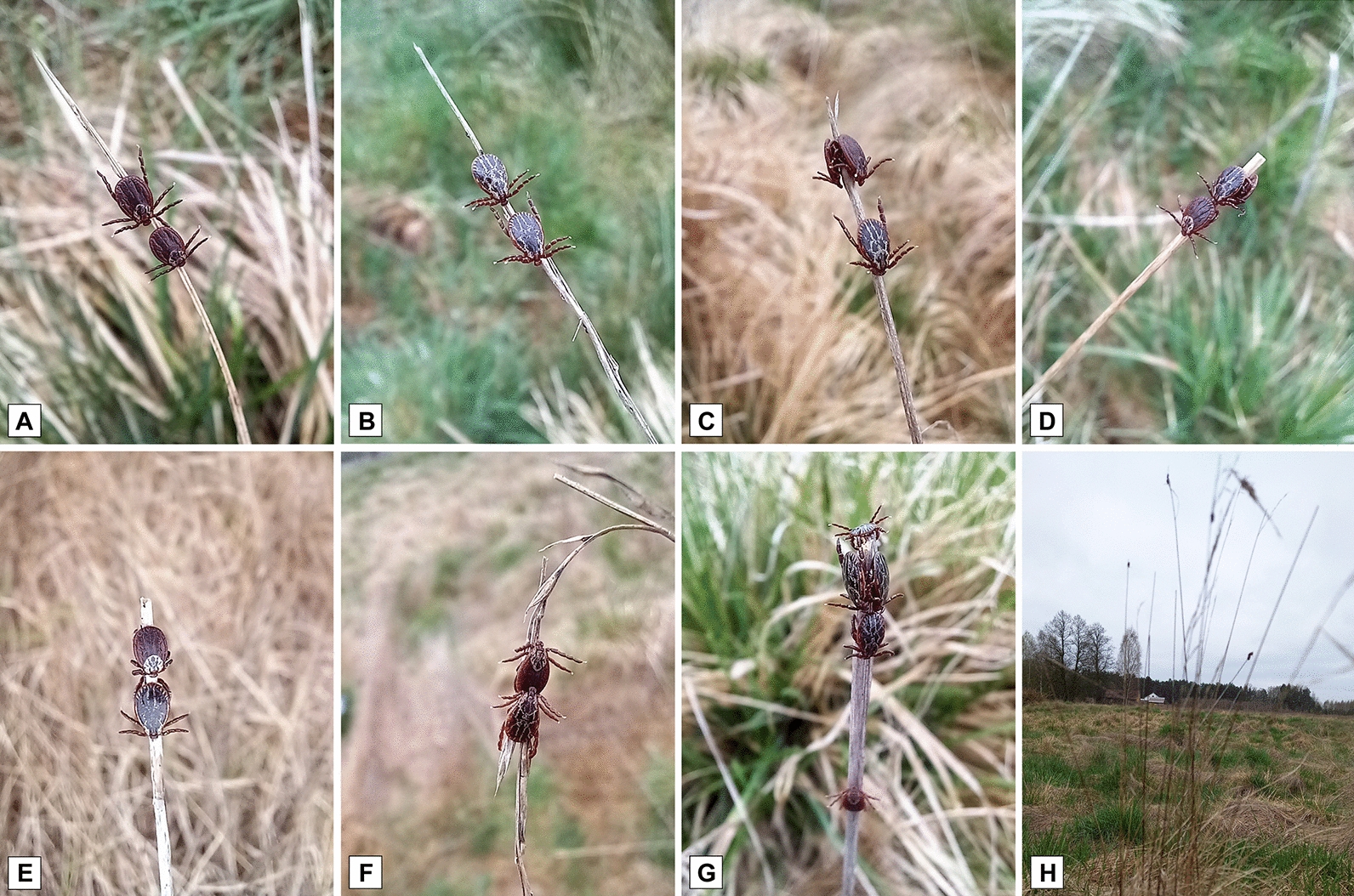
Clusters of adult questing *Dermacentor reticulatus* ticks on grass stems. **a** Two females, **b** 2 males, **c** 1 male and 2 females, **d** 1 female and 2 males, **e**, **f** 1 male and 1 female, **g** 4 males and 1 female, **h** 1 plant with 8 ticks on stems

A total of 371 *D. reticulatus* ticks (219 females, 152 males) were collected from 270 grass stems in the combined 1200-m-long transect. Additionally, 10 *I. ricinus* (7 females, 3 males) were found accompanying *D. reticulatus* ticks on stems (not analysed further). Individual plants with four, seven and 11 ticks spread over several stems were also observed (Fig. 2a–h).

**Fig. 3:**
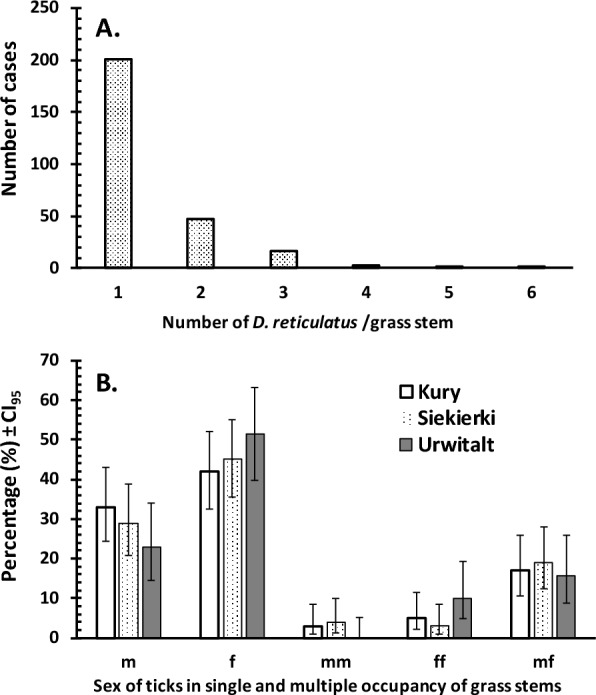
**a** Frequency distribution of single and multiple *D. reticulatus* tick occupancy on grass stems. The percentages of grass stems with 1-, 2-, 3-, 4-, 5- and 6-tick occupancy were 74.4%, 17.4%, 5.9%, 1.1%, 0.7% and and 0.4%, respectively (see also Table 1). **b** Percentage of grass stems occupied by the various combinations of *D. reticulatus* ticks: single male (m), single female (f), multiple tick occupancy–only males (mm), multiple tick occupancy–only females (ff) and multiple tick occupancy–mixed sexes (mf). The number of records at each of the 3 collection sites for m, f, mm, ff and mf assemblages was 33, 42, 3, 5 and 17, respectively, at Kury; 29, 45, 4, 3 and 19, respectively, at Siekierki; and 16, 36, 0, 7 and 11, respectively, at Urwitałt. CI_95_, 95% Confidence interval

The distribution of the number of *D. reticulatus* per grass stem is shown in Fig. 3a. The majority of grass stems were occupied by just a single individual tick (201/270, 74.4%), followed in frequency by two-tick clusters (the second most common category) (Fig. 3a; Table 1). Up to six *D. reticulatus* individuals were noted on one particular grass stem (Figs. 2, 3a).

**Table 1:** Percentage of female, male and mixed *Dermacentor reticulatus* ticks occupying a single stem of grass

Combination	Male	Female	Mix	Total
Single tick per blade	28.8	45.6	0	74.4
Two-tick clusters	2.6	5.2	9.6	17.4
Three-tick clusters	0	0.37	5.56	5.9
More than three-tick clusters	0	0	2.2	2.2

Analysis of the five possible combinations of tick occupancy on grass stems (single male, single female, ≥ 2 females, ≥ 2 males, ≥ 2 ticks of different sexes) revealed that there was no site effect for any of the 5 combinations (Fig. 3b): for site × single male,* χ*^2^_2_ = 2.06, *P* = 0.36; site × single female, *χ*^2^_2_ =  < 0.001, *P* > 0.99; site × multiple males,* χ*^2^_2_ = 4.42, *P* = 0.110; site × multiple females,* χ*^2^_2_ = 3.45, *P* = 0.178; site × mixed sexes,* χ*^2^_2_ = 0.061, *P* = 0.74). The site effect in the minimum sufficient model (MSM) (Table 2) arose because while the number of observations from Kury and Siekierki was 100 in each case, only 70 observations were recorded from Urwitałt

However, there were significant differences between each of the 10 possible two-way comparisons of the five combinations of tick occupancy on grass stems (Table 2). The percentages for each combination by site are shown in Fig. 3b where it can be seen how similar the pattern was at all three sites.

**Table 2:** Minimum sufficient statistical model for the five combinations of tick occupancy on grass stems

Combination of tick occupancy^a^	*χ*^2^	*df*	*P*
mm × ff	25.1	1	< 0.001
mm × mf	39.3	1	< 0.001
m × mm	46.0	1	< 0.001
f × mm	52.1	1	< 0.001
ff × mf	66.2	1	< 0.001
m × ff	79.8	1	< 0.001
f × ff	92.5	1	< 0.001
m × f	266.1	1	< 0.001
m × mf	163.1	1	< 0.001
f × mf	198.1	1	< 0.001
Site	6.96	2	0.031

For goodness of fit of the final model to the data: *χ*^2^_78_ = 12.81, *P* > 0.99

^a^*f* Single female,* ff* multiple tick occupancy–only females, *m* single male, *mf* multiple tick occupancy–mixed sexes, *mm* multiple tick occupancy–only males

While occupancy by a single tick accounted for most observations, cases of occupancy by a single female were more frequently observed compared to cases of occupancy by a single male (Fig. 3b). Multiple occupancy was less frequently recorded than single occupancy, and mixed-sex clusters were significantly more common than single-sex clusters (Fig. 3b; see also Table 2 for statistical analysis).

When data on study sites were combined, mixed-sex clusters accounted for 17.4% (95% CL: 13.86–21.57%) of observations, while for multiple male-only and multiple female-only clusters, the values were 2.6% (95% CL: 1.39–4.73%) and 5.6% (95% CL: 3.65–8.33%), respectively.

## Discussion

The key finding of our observational study is that mixed-sex clusters of *D. reticulatus* occur on grass stems more frequently than single-sex clusters, with the likely result of the simultaneous transfer of ticks of both sexes to a potential host following contact of the latter with the grass stem. Given that mating in this species occurs only after infestation of a new host, this finding can be interpreted as reflecting an important component of a successful breeding strategy of *D. reticulatus* ticks. Moreover, it suggests the involvement of off-host arrestment (assembly) pheromones (causing the cessation of tick kinetic activity, leading to clusters of individuals in their natural environment [3]) that partially resemble sex pheromones in terms of their function (i.e. attractiveness to the opposite sex [3]).

As a breeding strategy, the more frequent occurrence of mixed-sex clusters on grass stems depends on a combination of two behavioural features: formation of off-host clusters and recognition of tick sex that requires an exchange of information between tick individuals. By definition, sex pheromones are semiochemicals emitted by individuals of one sex that mediate the sexual behaviour of the opposite sex [3]. The first tick sex pheromone (2,6-dichlorophenol) was discovered in the lone star tick *Amblyomma americanum* over 50 years ago [25], but to the best of our knowledge, the pheromones that induce sex-related clustering of ticks off-host have not yet been identified. Nevertheless, in recent years, an impressive amount of novel knowledge has accumulated on the chemical ‘language’ (production and release of semiochemicals, information-transferring molecules) used by ticks for inter- and intra-species communication [3, 26–28].

The response to various chemical compounds involves a sequential recognition process, forming a hierarchy of behaviours that culminate in aggregation and sexual responses [2]. In ticks, these behaviours (i.e. formation of clusters, mate-finding and courtship) are regulated mostly by pheromones that induce certain behavioural responses in related organisms [2, 3, 28]. Different tick-borne semiochemicals are now known to induce off-host aggregation in the environment, on-host non-sexual aggregation, on-host sexual aggregation, copulation and ejaculation [2]. Off-host aggregation has been attributed to arrestment (= assembly) pheromones; on-host non-sexual aggregation has been attributed to attraction–aggregation–attachment (AAA) pheromones described in *Amblyomma* spp.; on-host sexual aggregation, copulation and ejaculation have been attributed to different kinds of sex pheromones [2, 3].

The phenomenon of multi-sex aggregation on grass stems observed in the current study can be a consequence of combined arrestment behaviour (ticks clustering in the environment) and sexual aggregation (mostly mixed-sex aggregation). Arrestment and assembly pheromones are prevalent among tick species, appearing in both hard (Ixodidae) and soft (Argasidae) ticks. The initiation of arrestment behaviour is primarily facilitated through contact with excretions from other ticks [2, 3], and although widespread among tick species, arrestment pheromones have not been reported in all tick species. For example, and in contrast to our finding, Taylor et al. [29] found no evidence of similar behaviour (off-host aggregation/assembly) in ticks of the genus *Dermacentor* that occur in the Nearctic region (*D. variabilis* and *D. andersoni)*.

Tick clustering behaviour seems to follow a two-step process: first, an attraction to a volatile source, and second, arrestment triggered by various purines. Despite purines having a very low vapor pressure and not being effective attractants, they do play a role in the second step of the clustering process [3]. Ammonia and perhaps other volatiles emanating from tick faeces gradually attract free-living, unfed adults and even nymphs to the point source. Contact with the purines (especially guanine and xanthine) triggers the arrestment response and causes ticks to cease activity, forming a cluster [3]. Formation of tick clusters in the environment may actually positively affect many aspects of tick life; for example clusters can enhance mating and host-finding success [3, 26]. In soft ticks, clustering behaviour is thought to confer survival advantages, as individuals within the cluster accumulate in locations that are conducive to avoiding adverse environmental conditions, such as desiccation. Additionally, these clustered sites are more likely to be encountered by potential hosts, enhancing the ticks’ chances of finding suitable hosts for feeding [3].

In prostriate ticks, such as *I. ricinus*, clusters of host-seeking ticks are often found on vegetation, which is believed to favour contact with passing hosts [3]. Clustering of *I. ricinus* and *Ixodes scapularis* on vegetation facilitates contact between the sexes. In a pioneering study, Graf [8] reported that up to 70% of *I. ricinus* females collected from vegetation had already mated, indicating that prostriate ticks can mate preprandially on the ground or on vegetation, in contrast to metastriate ticks which invariably mate only while attached to hosts. In the case of *Ixodes* males of nidicolous species, mating can only take place before attachment to hosts, hence only preprandially, since the males of these species never seek hosts. Other *Ixodes* species, such as those of the *I. ricinus* complex can mate once again on the surface of their hosts [2].

Perhaps the most interesting feature of this study is the repeatability of our observations of an almost identical pattern of aggregation (cluster structure) across our three study sites, which were not contiguous. Indeed, the sites are isolated from one another by distances varying from 50 to 200 km, and they differ in terms of vegetation cover (described in detail in [20, 30]). Despite this, the distribution of questing adult ticks on grass stems was very similar in each of our three sites, which is supportive of our research hypothesis. It would be interesting to conduct similar studies in other regions endemic for *D. reticulatus* (as well as on other tick species), including also the monitoring of temperature and humidity, to assess the generality of this phenomenon.

## Conclusions

Mixed-sex clusters of *D. reticulatus* ticks on grass stems were significantly more common than single-sex clusters, and given that this tick species mates only on newly infested hosts, such a strategy would inevitably result in the simultaneous transfer of both sexes from grass stem to host following contact of a suitable host with the supporting grass stem. The widespread occurrence of this expansive tick species and the repeatability of our observations indicate that mix-sex clusters may enhance the efficiency of mate finding and hence mating, and thereby reflect the efficient breeding strategy of *D. reticulatus* ticks.

## Data Availability

All relevant data are included in the article.
